# Distortion of the Major Histocompatibility Complex Class I Binding Groove to Accommodate an Insulin-derived 10-Mer Peptide*

**DOI:** 10.1074/jbc.M114.622522

**Published:** 2015-06-16

**Authors:** Chihiro Motozono, James A. Pearson, Evy De Leenheer, Pierre J. Rizkallah, Konrad Beck, Andrew Trimby, Andrew K. Sewell, F. Susan Wong, David K. Cole

**Affiliations:** From the ‡ Division of Infection and Immunity and; the ¶ Institute of Molecular and Experimental Medicine, Cardiff University School of Medicine, Heath Park, Cardiff CF14 4XN, United Kingdom,; the § Department of Immunology, Kinki University School of Medicine, Osaka 589–8511, Japan, and; the ‖ Cardiff University School of Dentistry, Heath Park, Cardiff CF14 4XY, United Kingdom

**Keywords:** beta cell, insulin, T-cell receptor (TCR), type 1 diabetes, x-ray crystallography, NOD mouse, pMHC

## Abstract

The non-obese diabetic mouse model of type 1 diabetes continues to be an important tool for delineating the role of T-cell-mediated destruction of pancreatic β-cells. However, little is known about the molecular mechanisms that enable this disease pathway. We show that insulin reactivity by a CD8^+^ T-cell clone, known to induce type 1 diabetes, is characterized by weak T-cell antigen receptor binding to a relatively unstable peptide-MHC. The structure of the native 9- and 10-mer insulin epitopes demonstrated that peptide residues 7 and 8 form a prominent solvent-exposed bulge that could potentially be the main focus of T-cell receptor binding. The C terminus of the peptide governed peptide-MHC stability. Unexpectedly, we further demonstrate a novel mode of flexible peptide presentation in which the MHC peptide-binding groove is able to “open the back door” to accommodate extra C-terminal peptide residues.

## Teaser

**Background:** CD8^+^ T-cells play a central role in type 1 diabetes (T1D) by recognizing insulin peptides displayed by MHC.

**Results:** A novel flexible MHC binding mode accommodates extra C-terminal peptide residues.

**Conclusion:** Unusual peptide-MHC binding might explain weak TCR affinity of a natural T1D epitope.

**Significance:** MHC peptide binding can be highly flexible around the F-binding pocket.

## Introduction

Type 1 diabetes (T1D)^7^ is an autoimmune disease affecting children and young adults where CD8^+^ T-cells have recently been shown to play a central role in pancreatic β-cell destruction (1–7). A number of CD8^+^ T-cell T1D epitopes from the key autoantigenic target proinsulin have been identified (6, 8, 9). How these autoreactive CD8^+^ T-cells escape thymic selection and cause pathology in the periphery is still under debate. However, some evidence suggests that the nature of the interaction between the clonally expressed T-cell receptor (TCR) and self-peptide-major histocompatibility complex class I (pMHCI) may drive this selection. The strength and/or duration of binding between the TCR and pMHCI (10), as well as different mechanical forces (11), can determine the threshold of T-cell activation. Accumulated data suggest that most self-reactive T-cells express TCRs that interact weakly with pMHC compared with pathogenic T-cells (5, 10, 12, 13). These observations are compounded by the low stability, predicted or demonstrated, for many autoimmune-pMHC interactions (5, 14–18). Other molecular investigations of T-cell-induced autoimmunity have also demonstrated suboptimal TCR binding through atypical TCR conformation, compared with most pathogen-specific TCRs (17, 19–21). These factors have previously been considered to be the basis for poor negative selection of autoreactive T-cells, which may escape from the thymus and become activated in the periphery, potentially through molecular mimicry (22, 23), and thence induce autoimmunity.

The non-obese diabetic (NOD) mouse model, which develops spontaneous diabetes, has been widely used for investigating T1D (24–27). There are many parallels between T1D in humans and NOD mice, and findings in these mice have paved the way for important discoveries in humans (27, 28). In NOD mice, in which both genetic susceptibility and environment play a role in disease development, both CD4^+^ and CD8^+^ T-cells, recognizing a number of different autoantigens (reviewed in Ref. 29) are important in autoimmune attack on pancreatic islet β-cells. We have previously cloned a diabetogenic CD8^+^ T-cell (G9C8) from the islets of young prediabetic NOD mice, which lyses islets *in vitro* and causes diabetes within 5–10 days after transfer to young non-diabetic NOD mice and NOD.scid mice (24, 30). The G9C8 T-cell clone recognizes insulin B chain amino acids 15–23, and T-cells reacting to this epitope can be highly represented in the small number of cells in the early infiltrate (8), although other specificities become more dominant later. It has been shown that epitopes within the insulin B chain have a prime role in the development of T1D, because substitution at position 16 of the B chain abolishes CD4^+^ (31) and CD8^+^ T-cell reactivity (8, 32). This region of the insulin B chain has also been identified as an important autoantigen in humans (26, 33, 34), offering an important model system for investigating the human form of the disease.

Here, we used cellular and biophysical methods to investigate the molecular interaction between the G9C8 TCR and the native insulin B chain 10-mer peptide, ^15^LYLVCGERGF^24^ (G9GF) and 9-mer peptide, ^15^LYLVCGERG^23^ (G9G) as well as a heteroclitic form of the peptide, LYLVCGER**V** (G9V), presented by H-2K^d^. G9V was designed to improve MHC stability and has been shown to activate G9C8-like T-cells more strongly than the native G9G peptide (32), although the molecular basis for this increased potency has not been fully resolved. We solved the atomic structures of each of the peptides in complex with H-2K^d^, demonstrating the peptide residues that interact with the MHC binding groove and identifying the solvent-exposed residues that are most likely to contact the TCR. These data provide the first molecular insight into CD8^+^ T-cell-induced β-cell destruction via recognition of the insulin B chain in this important disease model of T1D and demonstrate a novel flexible peptide-MHC binding mode that has broad implications for T-cell antigen presentation.

## Experimental Procedures

### 

#### 

##### CD8 T-cells

Insulin-reactive CD8^+^ T-cells (G9C8) were isolated from spleen cells from 5–8-week-old transgenic G9Cα^−/−^ NOD mice (30).

##### [^3^H]Thymidine Incorporation Proliferation Assay

Splenic CD8^+^ T-cells were purified using a Miltenyi MACS CD8^+^ isolation kit (>90% purity) and cultured at 10:1 with bone marrow-derived dendritic cells with the LYLVCGERGF (G9GF), LYLVCGERG (G9G), or LYLVCGERV (G9V) peptide in RPMI medium supplemented with 5% FCS, 2 mm l-glutamine, 0.05 mm 2-mercaptoethanol, penicillin/streptomycin. Each sample was plated in duplicate. After 48 h of incubation, cells were pulsed with 0.5 μCi of [^3^H]thymidine for 18 h, harvested, and counted to determine [^3^H]thymidine incorporation.

##### ELISAs for Chemokine and Cytokine Production

Supernatants were removed from the proliferation assay cultures prior to the addition of [^3^H]thymidine. MIP1β was measured by sandwich ELISA (R&D systems), whereas IFNγ was measured using a similar protocol (BD Biosciences) with the modification that the capture antibody was diluted in carbonate buffer and incubated at 4 °C overnight. Plates were blocked at 37 °C for 1 h, and the detection antibody was incubated for 1 h at room temperature.

##### Staining of Insulin-specific CD8 T-cells with H-2K^d^·Peptide Tetramers

Splenocytes from 6-week-old G9Cα^−/−^ NOD mice were isolated, and red cells were lysed. 1 × 10^6^ splenocytes were then preincubated with 50 nm dasatinib (Axon Medchem) for 30 min at 37 °C, and cells were washed in PBS with 2% FCS and stained for 15 min at 37 °C using 0.5 μg of each of the H-2K^d^·peptide tetramers (National Institutes of Health tetramer facility): AYAAAAAAV (negative control), G9GF, G9G, or G9V. Cells were then washed again prior to the addition of CD8α FITC (clone 53-6.7, BD Biosciences), CD4 PE-Cy7 (clone RM4-5, eBioscience), CD19 PerCpCy5.5 (clone 1D3, eBioscience), CD11b BV421 (clone M1/70, Biolegend) and checked for viability using an eFluor 780 viability dye (eBioscience). Cells were incubated at 4 °C for 30 min prior to washing again before acquisition on a BD Biosciences FACSCanto II, with data analyzed with Flowjo version 7.6.5 software (Treestar) gating on Live CD8^+^CD19^−^CD11b^−^CD4^−^Tetramer^+^ T-cells. The mean fluorescence intensity was then calculated and further analyzed using GraphPad Prism version 4 software.

##### Construct Design

The TCRα and -β chains and the H-2K^d^ heavy chains (tagged and untagged with a biotinylation sequence) and the human β2m chain were generated by PCR mutagenesis (Stratagene) and PCR cloning. All sequences were confirmed by automated DNA sequencing (Lark Technologies). The G9C8 TCRα and -β chains, the H-2K^d^ heavy chains (residues 1–248) (α1, α2, and α3 domains), and β2m (residues 1–100) were also cloned. G9C8 TCRα and -β chains, the H-2K^d^ α chains, and β2m sequences were inserted into separate pGMT7 expression plasmids under the control of the T7 promoter (35).

##### Protein Expression, Refolding, and Purification

Competent Rosetta DE3 *Escherichia coli* cells were used to produce the G9C8 TCRα and -β chains, the H-2K^d^ heavy chains, and β2m in the form of inclusion bodies using 0.5 mm isopropyl 1-thio-β-d-galactopyranoside to induce expression, and proteins were chemically refolded as described previously (36).

##### pMHCI Biotinylation

Biotinylated pMHCI was prepared as described previously (37).

##### pMHC Stability Assays

Thermal stability of H-2K^d^ complexes was assessed by circular dichroism (CD) spectroscopy, monitoring the change in ellipticities at 218 nm. Data were collected on an Aviv Model 215 spectropolarimeter (Aviv Biomedical Inc., Lakewood, NJ) using a 0.1-cm quartz cell. Proteins were dissolved in PBS at concentrations of 2.5 μm. Melting curves were recorded in 0.5 °C intervals from 4 °C up to a maximum temperature of 90 °C when protein aggregation was observed. Melting curves were analyzed assuming a two-state trimer-to-monomer transition from the native (N) to unfolded (U) conformation N_3_ ↔ 3U with an equilibrium constant *K* = [U]^3^/[N_3_] = *F*/(3*c*^2^(1 − *F*)^3^), where *F* and *c* are the degree of folding and protein concentration, respectively. Data were fitted as described (38). Fitted parameters were the melting temperature (T*_m_*), van't Hoff's enthalpy (Δ*H*_vH_), and the slope and intercept of the native baseline. Because all protein complexes aggregated upon unfolding, the ellipticity of the unfolded state was set as a constant of −4,500 degrees cm^2^ dmol^−1^ (39, 40).

##### Surface Plasmon Resonance Analysis

Binding analysis was performed using a BIAcore 3000^TM^ equipped with a CM5 sensor chip as described previously (41). Binding analysis was performed four times in independent experiments using pMHC monomers generated in house and from the National Institutes of Health tetramer facility. Approximately 200–500 RU of peptide-H-2K^d^ (in complex with G9GF, G9G, or G9V) was attached to the CM5 sensor chip at a slow flow rate of 10 μl/min to ensure uniform distribution on the chip surface. Combined with the small amount of peptide-H-2K^d^ bound to the chip surface, this reduced the likelihood of off-rate-limiting mass transfer effects. The G9C8 TCR was purified and concentrated to ∼140 μm on the same day of surface plasmon resonance analysis to reduce the likelihood of TCR aggregation affecting the results. For equilibrium analysis, eight serial dilutions were prepared in triplicate for each sample and injected over the relevant sensor chips at 25 °C. TCR was injected over the chip surface using kinetic injections at a flow rate of 45 μl/min using H-2K^d^·AYAAAAAAV or HLA-A*0201·ALWGPDPAAA in different experiments as negative controls.

##### Crystallization, Diffraction Data Collection, and Model Refinement

All protein crystals were grown at 18 °C by vapor diffusion via the sitting drop technique. 200 nl of each pMHCI (10 mg/ml) in crystallization buffer (10 mm Tris, pH 8.1, and 10 mm NaCl) was added to 200 nl of reservoir solution. H2K^d^-G9GF crystals were grown in 4% PEG 4000, 0.1 m sodium acetate, pH 4.6, H-2K^d^·G9G crystals were grown in 20% PEG 3350, 0.2 m sodium malonate, 0.1 m Bistris propane, pH 6.5, and H-2K^d^·G9V crystals were grown in 20% PEG 6000, 0.2 m calcium chloride, 0.1 m Tris propane, pH 8.0 (42). All crystals were soaked in 30% ethylene glycol before cryo-cooling. All crystallization screens and optimization experiments were completed using an Art-Robbins Phoenix dispensing robot (Alpha Biotech Ltd., UK). Data were collected at 100 K at the Diamond Light Source (Oxfordshire, UK). All data sets were collected at a wavelength of 0.98 Å using an ADSC Q315 CCD detector. Reflection intensities were estimated with the XIA2 package (43), and the data were scaled, reduced, and analyzed with SCALA and the CCP4 package (44). Structures were solved with molecular replacement using PHASER (45). Sequences were adjusted with COOT (46), and the models were refined with REFMAC5. Graphical representations were prepared with PyMOL (47). The reflection data and final model coordinates were deposited in the Protein Data Bank (H-2K^d^·G9GF, code 4Z78; H-2K^d^·G9G, code 4WDI; and H-2K^d^·G9V, code 4Z76).

## Results

### 

#### 

##### Insulin-reactive CD8 T-cells Are Stimulated by Native and Altered Insulin Peptides

**FIGURE 1.**
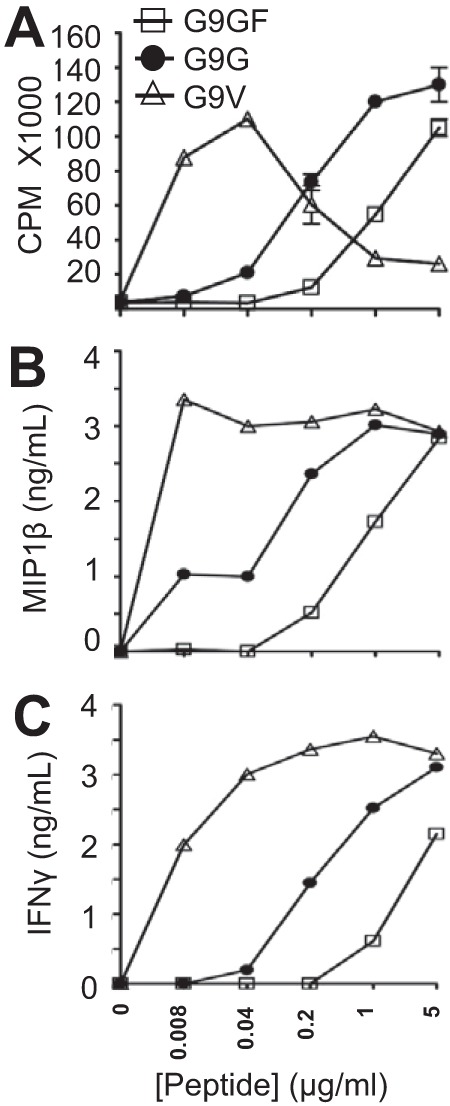
**T-cell functional assays.** Purified insulin-reactive CD8^+^ T-cells were incubated for 72 h with native 10-mer peptide, LYLVCGERGF (G9GF), native 9-mer peptide, LYLVCGERG (G9G), or heteroclitic peptide, LYLVCGERV (G9V), together with bone marrow-derived dendritic cells. [^3^H]Thymidine was added, and incorporation was measured upon harvesting 18 h later and counting on a micro-β-counter to measure T-cell proliferation (*A*). Supernatants from the cultures were removed prior to the addition of [^3^H]thymidine and used to measure MIP1β (*B*) and IFNγ (*C*) by ELISA.

We have previously demonstrated that the G9C8 T-cell clone can induce rapid onset T1D in NOD.scid mice (24). This pathology is governed by the ability of G9C8 T-cells to recognize a region of the insulin B chain protein that is conserved between humans and mice and is an autoantigen in both species (26, 33, 34). The G9C8 T-cell clone recognized both the native 9-mer (G9G) and native 10-mer (G9GF) versions of this peptide but generated a stronger response (proliferation, MIP1β, and IFNγ production) to the G9G peptide compared with the G9GF peptide (Fig. 1). The G9C8 T-cell clone was about 5 times more sensitive to the G9G peptide compared with G9GF in all assays. Interestingly, substitution of Gly for Val at residue 9 in the G9V peptide, distal from the central bulge of the peptide that is usually involved in TCR contacts, increased activation markedly compared with the G9G peptide (the G9C8 T-cell clone was at least 5 times more sensitive to G9V compared with G9G in all assays) (Fig. 1). These observations warranted further investigation of the molecular rules that govern recognition of this important autoantigen during T1D.

##### Recognition of the Native G9G Peptide Is Characterized by Weak TCR Binding

**FIGURE 2.**
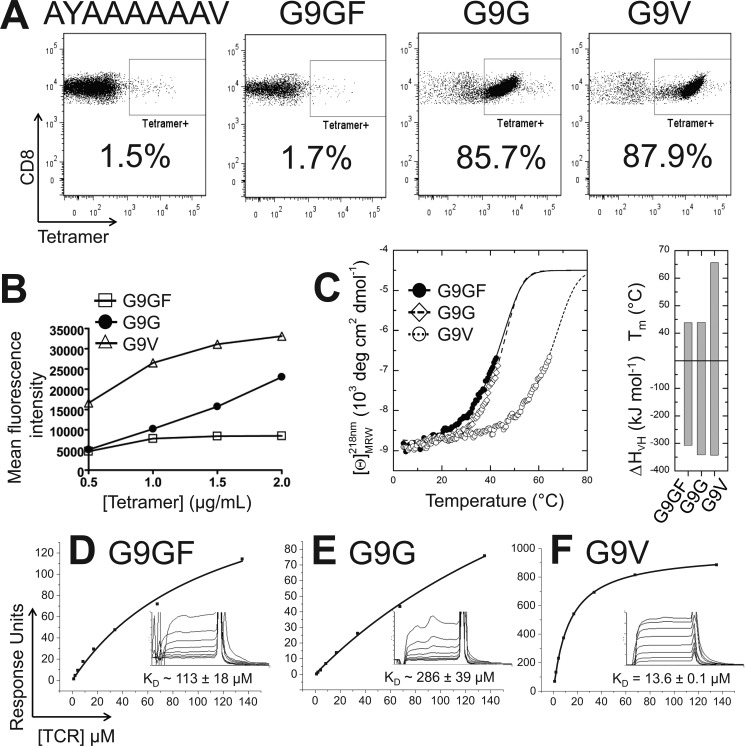
**Molecular characterization of G9C8 T-cell antigen recognition.** *A*, staining of insulin-reactive CD8^+^ T-cells with peptide-tetramer complexes. Purified insulin-reactive CD8^+^ T-cells from the NOD mouse were incubated with H-2K^d^·AYAAAAAAV negative control tetramer, H-2K^d^·G9GF tetramer, H-2K^d^·G9G tetramer, and H-2K^d^·G9V tetramer, followed by anti-CD8 monoclonal antibody, and analyzed by flow cytometry. *B*, the mean fluorescence intensity of the tetramer staining is shown for different concentrations of each tetramer. *C*, CD thermal denaturation curves recorded at 218 nm are shown for selected pMHC samples. *Dots*, measured values fitted assuming a two-state trimer-to-monomer transition as described under “Experimental Procedures.” The *panel* to the *right* shows bar graphs of the thermal stability with respect to melting temperature (*top*) and van't Hoff's enthalpy of unfolding (*bottom*). *D–F*, binding affinity of the G9C8 TCR interaction at 25 °C. Eight serial dilutions of the G9C8 TCR were measured; representative data from four independent experiments are plotted. Binding analysis was performed using pMHC monomers generated in house and from the National Institutes of Health tetramer facility. The equilibrium binding constant *K_D_* values were calculated using a nonlinear curve fit (*y* = (P_1_*x*)/P_2_ + *x*); mean plus S.D. values are shown. In order to calculate each response, the G9C8 TCR was also injected over a control sample (H-2K^d^·AYAAAAAAV or HLA-A*0201-ALWGPDPAAA in different experiments) that was deducted from the experimental data (shown in the *inset*). *D*, G9C8 *versus* H-2K^d^·G9GF. *E*, G9C8 *versus* H-2K^d^·G9G. *F*, G9C8 *versus* H-2K^d^·G9V.

**FIGURE 3.**
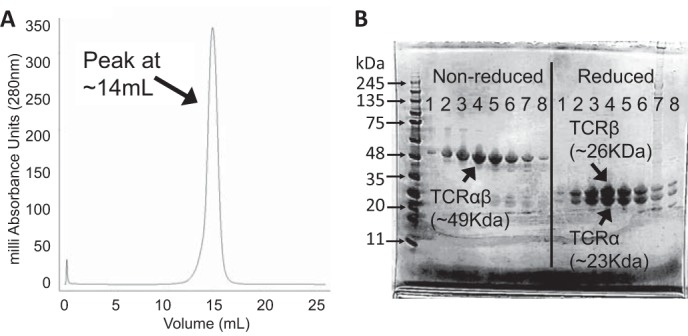
**G9C8 protein purification and analysis.** *A*, gel filtration (size exclusion) using an S200 Superdex 25-ml bed volume column. A symmetrical peak at 14 ml (the expected elution profile for heterodimeric αβ TCR) was observed. *B*, SDS gel analysis of fractions corresponding to 1-ml sample collections from 11–18 ml (*numbered 1–8* on the gel) from the corresponding gel filtration shown in *A*. Molecular weight markers with corresponding protein sizes are shown in the *first lane*. The non-reduced gel shows a single band of protein at ∼49 kDa, corresponding to the expected size of the G9C8 αβ TCR heterodimer. To ensure that both chains were present, we performed a reducing gel. Equal amounts of two protein species at ∼23 and 26 kDa were observed, corresponding to the expected size of the G9C8 TCRα and -β chains, respectively.

We investigated the molecular interaction between the G9C8 TCR and the different peptide ligands by performing tetramer staining experiments. Although the G9GF peptide induced a low level of T-cell activation, the H-2K^d^·G9GF tetramers did not robustly stain the G9C8 T-cell clone (Fig. 2*A*). The H-2K^d^·G9V tetramer stained 87.9% of the G9C8 clone, in line with the strong activation observed with this ligand (Fig. 2*A*). Although the H-2K^d^·G9G tetramer stained more weakly compared with H-2K^d^·G9V, consistent with the T-cell activation analysis, the level of staining was still high (85.7% G9C8 clone staining). The biggest difference between the H-2K^d^·G9G and H-2K^d^·G9V tetramers was the mean fluorescence intensity, being substantially higher for H-2K^d^·G9V (Fig. 2*B*). In order to further examine the strong staining of the H-2K^d^·G9G tetramer compared with H-2K^d^·G9GF, we determined the thermal stability of the soluble pMHC proteins using CD spectroscopy. Consistent with our previous findings (32), the lack of an optimal anchor at the C terminus of the G9GF and G9G peptides had a large negative effect on their stability compared with G9V, which showed a melting temperature over 20 °C higher than G9G and G9GF (Fig. 2*C*). Similar observations from other groups have been reported in which modification of the N-terminal positions of a melanoma peptide increased pMHC stability and immunogenicity (48). The similarly low thermal stability of both G9GF and G9G did not reveal an obvious mechanism for the high level of H-2K^d^·G9G tetramer staining compared with H-2K^d^·G9GF, although G9GF was slightly less stable than G9G overall. Thus, we performed surface plasmon resonance (Fig. 2, *D* and *E*) using recombinant soluble G9C8 TCR (Fig. 3) injected over a sensor chip coated with H-2K^d^·G9GF, H-2K^d^·G9G, and H-2K^d^·G9V. The G9C8 TCR bound to the non-native H-2K^d^·G9V with a comparatively strong affinity (*K_D_* = 13.6 μm). This was in contrast to the weaker binding affinity to the native G9GF and G9G peptides (*K_D_* ∼113 and ∼286 μm, respectively), mirroring the effect on TCR affinity by altering peptide anchor residues reported before (49, 50). These data further confounded the enhanced T-cell activation and tetramer staining of the G9G peptide compared with G9GF, because the G9C8 TCR bound to H-2K^d^·G9GF with more than 2-fold stronger affinity compared with H-2K^d^·G9G. However, because the G9GF and G9G peptides formed relatively unstable pMHC complexes and because the TCR affinity was weak, these affinities should be considered reproducible estimates rather than absolute values. As such, the tetramer staining and T-cell activation assays probably represent a more accurate comparative estimation of the affinity differences between the G9C8 TCR and the G9G/G9GF peptides.

##### Peptide Residues Glu-7 and Arg-8 Extend Out of the MHC Binding Groove for Potential TCR Contact

**FIGURE 4.**
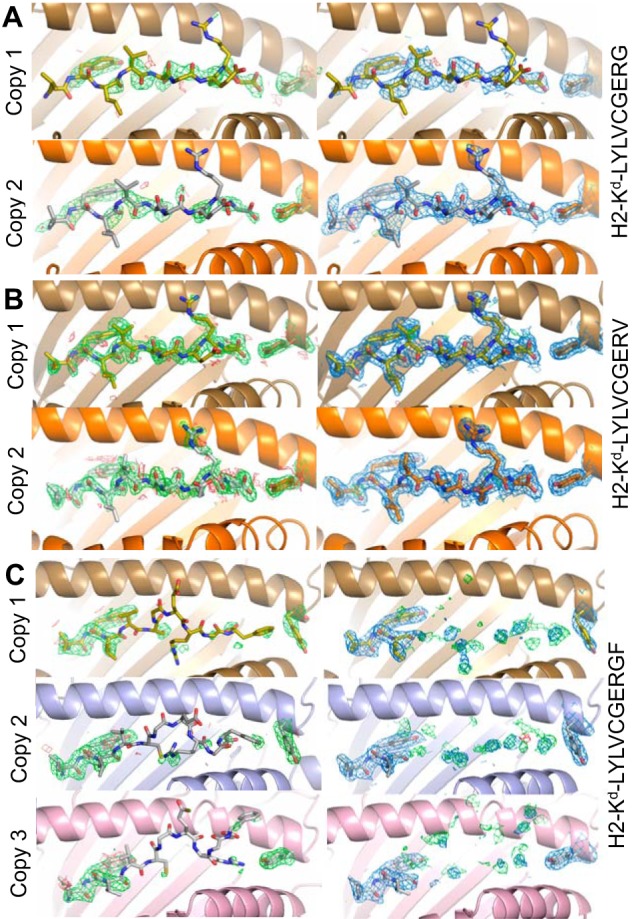
**Omit map and density plot analysis.** The *left column* shows omit maps in which the model was refined in the absence of the peptide and Tyr-84. Difference density is contoured at 3.0σ, positive contours are shown in *green*, and negative contours are *red*. The *right column* shows the observed map at 1.0σ after subsequent refinement using automatic non-crystallographic symmetry restraints applied by REFMAC5. *A*, the model for G9G, which has two identical copies of the pMHC motif in the asymmetric unit. Copy 1 is shown in *gold*, and copy 2 is *orange. B*, the model for G9V, which has two identical copies of the pMHC motif in the asymmetric unit. Copy 1 is shown in *gold*, and copy 2 is *orange. C*, the model for G9GF, which has three copies of the pMHC motif in the asymmetric unit. Peptide residues 1–4 are shown in the observed map (*right*). Copy 1 is shown in *gold*, copy 2 is *light blue*, and copy 3 is *light purple*. Note that the observed density (*marine blue*), has positive difference density close to the half-occupancy residues 4–10 and little negative density in the same region. The obvious disorder in the C-terminal 7 residues of the peptides does not extend to Tyr-84 in any of the copies.

**FIGURE 5.**
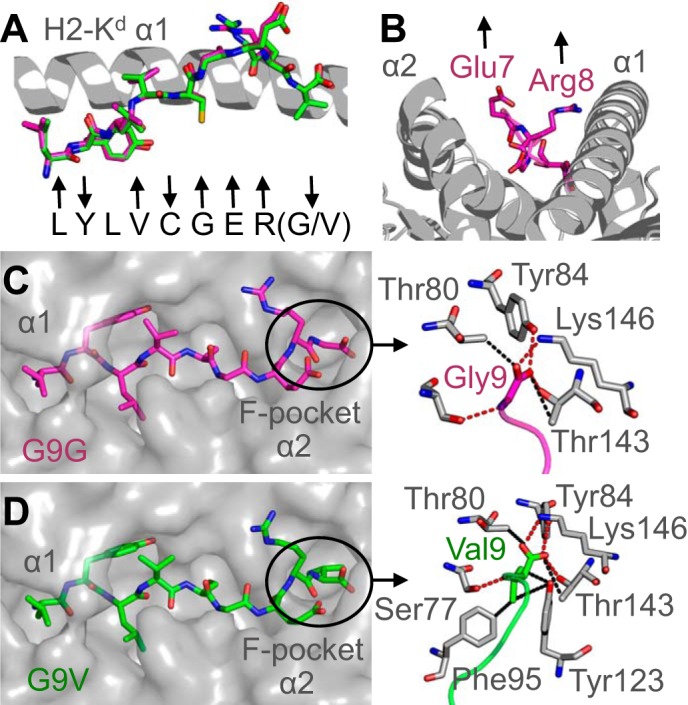
**Interactions between the peptide C terminus and the MHC binding groove determine pMHC stability.** *A*, superposition of the G9G peptide (*magenta sticks*) and G9V peptide (*green sticks*). The H-2K^d^ α1 domain is shown in a *gray schematic. Arrows below* the peptides indicate whether each residue is positioned away from the binding groove for potential TCR contact (*up arrow*), a primary or secondary anchor (*down arrow*), or in between (*no arrow*). *B*, peptide residues Glu-7 and Arg-8 (*magenta sticks*) bulge furthest away from the MHC groove (*gray schematic*). *C*, H-2K^d^ binding groove is shown in a *gray surface representation* demonstrating the extended conformation of the G9G peptide (*magenta sticks*). *Right*, the interactions between the C terminus of the G9G peptide (*magenta sticks*) and the MHC F-pocket (*gray sticks*). *D*, the H-2K^d^ binding groove is shown in a *gray surface representation* demonstrating the extended conformation of the G9V peptide (*green sticks*). *Right*, interactions between the C terminus of the G9V peptide (*green sticks*) and the MHC F-pocket (*gray sticks*).

In order to further understand the mechanism underlying the weak affinity between the G9C8 TCR and H-2K^d^·G9G, we solved the crystal structures of H-2K^d^·G9GF, H-2K^d^·G9G, and H-2K^d^·G9V. H-2K^d^·G9G crystallized in space group P1, and H-2K^d^·G9V crystallized in two distinct space groups, P1 and P1 21 1 (only the P1 data set is shown here in detail), which showed identical features (data not shown). H-2K^d^·G9GF crystallized in space group P 21 21 21 with three copies in the asymmetric unit (omit maps and density plots are shown in Fig. 4). All structures were determined to resolutions between 1.9 and 2.3 Å with crystallographic *R*_work_/*R*_free_ ratios within accepted limits as shown in the theoretically expected distribution (51) (Table 1). Alignment of the three structures generated route mean square deviation values of 0.491 (G9G *versus* G9GF), 0.355 (G9G *versus* G9V), and 0.647 (G9V *versus* G9GF), demonstrating that the overall conformation of all of the structures was very similar. The G9G and G9V structures featured unambiguous density around the peptides (Fig. 4, *A* and *B*), which were presented in an extended conformation, primarily anchored at peptide residues 2 and 9, with Cys-5 acting as a secondary anchor in the center of the peptide and residues 6–8 extending away from the groove (Fig. 5, *A* and *B*). For G9GF, clear electron density was only observed for peptide residues 1–4 (Fig. 4*C*), indicating flexibility in the rest of the peptide. Indeed, although all three copies were identical at the N-terminal end of the peptide, in copies 1 and 2, the peptide appeared to be anchored mainly at position 9, with position 10 extending out toward the end of the MHC groove and performing a secondary anchoring role (G9GF-stretched) (Fig. 4*C*). In copy 3, the peptide appeared to be anchored at position 10 (G9GF-bulged) (Fig. 4*C*). Although unambiguous density was not observed for peptide residues 5–8 in the H-2K^d^·G9GF structure, these residues were modeled in the same position and orientation as in the H-2K^d^·G9G and G9V structures, guided by agreement with the final model, which indicated no negative density for these residues in this conformation. The solvent-exposed nature of peptide residues 1, 4, 6, and 7 in the H-2K^d^·G9G and G9V structures (and possibly the G9GF structure) makes them the most likely TCR contact residues, supported by our previous data showing that modifications at peptide residues 1, 4, 6 (residue 6 could affect the conformation of residue 7), and 8 reduced T-cell recognition and could act as antagonists (32, 52). It is less likely that modification of these residues would affect peptide stability because of their apparent minimal role as anchor residues.

**TABLE 1:** **Data collection and refinement statistics for pMHC structures** One crystal was used for solving each structure. Values in parenthesis refer to the highest resolution shell. Root mean square deviation targets are automatically assigned by REFMAC5 according to the appropriate level based on the maximum likelihood method: 0.019 Å for bond lengths and 1.94° for bond angles. r.m.s., root mean square.

	H-2K^d^·G9GF	H-2K^d^·G9G	H-2K^d^·G9V
Protein Data Bank code	4Z78	4WDI	4Z76

**Data collection**			
Space group	P21 21 21	P1	P1
Cell dimensions			
*a*, *b*, *c* (Å)	46.2, 151.6, 182.2	46.9, 62.7, 72.7	48.0, 62.4, 72.3
α, β, γ (degrees)	90, 90, 90	68.1, 85.8, 85.2	69.8, 85.8, 87.1
Resolution (Å)	37.1-2.3	29.5-2.3	38.3-1.9
*R*_merge_ (%)	12.7 (92.9)	6.8 (35.1)	5.7 (27.2)
Mean *I*/σ*I*	10.4 (3.0)	8.6 (92.0)	8.6 (2.0)
Completeness (%)	100.0 (100.0)	90.2 (91.5)	89.9 (89.9)
Redundancy	7.2 (7.3)	2.1 (2.2)	2.1 (2.2)

**Refinement**			
Resolution (Å)	2.3 (2.36-2.30)	2.3 (2.37-2.31)	1.9 (1.93-1.88)
No. of reflections	57,725 (4,165)	28,604 (2,261)	54,089 (4,245)
No. of reflections in *R*_free_ set	2,925	1,527	2,886
*R*_work_/*R*_free_	18.8/23.3	20.6/28.3	18.6/22.9
Mean *B* value (Å^2^)	39.8	37.9	27.8
Overall coordinate error (Å)	0.174	0.235	0.191
r.m.s. deviations			
Bond lengths (Å)	0.015	0.016	0.015
Bond angles (degrees)	1.744	1.598	1.773

##### Interactions between the Peptide C Terminus and the MHC Binding Groove Determine pMHC Stability

**FIGURE 6.**
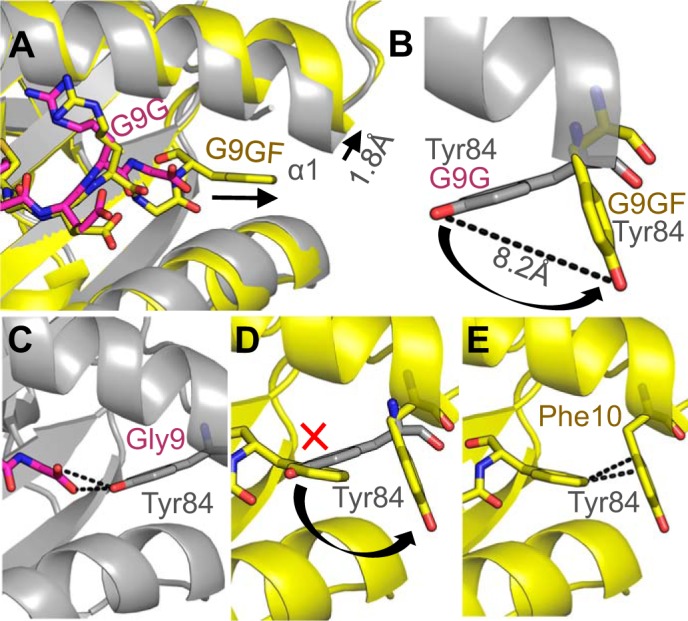
**MHC “opens the back door” to accommodate the extra C-terminal residue in the G9GF peptide.** *A*, superposition of the G9G peptide (*magenta sticks*) and the G9GF peptide (*yellow sticks*) showing the extended position of the G9GF-stretched C terminus and the movement in the H-2K^d^ α1 domain (G9G (*gray schematic*) and G9GF (*yellow schematic*)). *B*, MHC residue Tyr-84 “swings” 8.2 Å in G9GF-stretched (*yellow sticks*) complex compared with the G9G (*gray sticks*) complex. *C*, interaction between G9G residue Gly-9 (*magenta sticks*) and MHC residue Tyr-84 (*gray sticks*). *D*, MHC residue Tyr-84 would cause a steric clash with G9GF residue Phe-10 (*yellow sticks*) when positioned as in the G9G complex structure. *E*, interaction between G9GF residue Phe-10 (*yellow sticks*) and MHC residue Tyr-84 (*yellow sticks*).

We next investigated the interactions between the different peptides and H-2K^d^ (Table 2). G9G made 17 vdWs and 4 HBs (Fig. 6*C*), and G9V made 21 vdWs and 4 HBs (Fig. 6*D*), both through peptide residue 9. Although G9G only lost four vdW contacts with anchor residue Gly-9 compared with G9V (anchor residue Val-9), there were knock-on effects at the N terminus of the peptide (Table 2). The first 5 residues of G9V made 102 vdWs and 12 HBs with the MHC binding groove, whereas the first 5 residues of G9G only made 102 vdWs and 9 HBs (Table 2). These observations demonstrate the importance of optimal anchoring at both the N and C terminus of the peptide and help explain the substantially lower thermal stability of the G9G and G9GF peptides compared with G9V (Fig. 2*C*).

**TABLE 2:** **Peptide-MHC contact table** SB, salt bridge; BSA, buried surface area between the peptide and MHC; r.m.s. deviation, root mean square deviation calculated by aligning each pMHC complex (α-chain, peptide, and β2m) in PyMOL. A 3.4 Å cut-off was used for HBs and salt bridges, and a 4 Å cut-off was used for vdWs.

Peptide	H-2K^d^·G9G		H-2K^d^·G9V	
	vdW	HB/SB	vdW	HB/SB
Leu-1	27	2	28	3
Tyr-2	21	3	24	5
Leu-3	18	2	19	2
Val-4	13	1	12	1
Cys-5	25	1	19	1
Gly-6	6	2	6	2
Glu-7	12	3	14	3
Arg-8	19	1	18	1
Gly/Val-9	17	4	21	4
Total	158	19	161	22
BSA (Å^2^)	1,909.4		1,983.4	
r.m.s. deviation	G9G/G9V	0.355	G9V/G9GF	0.647
	G9G/G9GF	0.491		

The dynamic nature of the G9GF peptide, evident from the lack of electron density for the central and C-terminal portion of the peptide, made analysis of peptide-MHC contacts unreliable. However, this instability, presumably mediated by the extra residue in the G9GF peptide, could contribute toward the lower relative stability of the H-2K^d^·G9GF protein as well as altering interactions with the G9C8 TCR.

##### Tyr-84 in the MHC α1 Domain Swings Open Possibly to Enable Unusual Presentation of the G9GF Peptide

We expected that the additional residue at the C terminus in the G9GF peptide compared with G9G would require the central portion of the G9GF peptide to bulge further out of the groove to accommodate the extra residue, forcing G9GF into a completely different conformation compared with G9G, as is usually seen with longer MHCI-restricted peptides (53–55). Although the electron density was not definitive, our analysis indicated that the G9GF-bulged model anchored at position 10 mediating a slightly different conformation around peptide residues 6–8. However, in the more dominant conformation observed in the other two copies in the asymmetric unit (G9GF-stretched), the C terminus of the MHC α1 helix flexed by 1.8 Å, allowing Phe-10 to slide further down into the opening of the groove (Fig. 6*A*). Additionally, MHC residue Tyr-84 side chain underwent a large movement of 8.2 Å to swing out of the way of Phe-10, compared with its position in the G9GF-bulged, G9G, and G9V structures (Fig. 6*B*). These movements also altered the shape of the MHC F-pocket. Tyr-84 made three vdWs and one HB with the peptide in the G9G structure (Fig. 6*C*) but in that position would cause a steric clash with Phe-10 in the G9GF structure (Fig. 6*D*). In its alternative position in the G9GF-stretched structure, Tyr-84 could potentially form stabilizing interactions, including CH-π (π edge-to-face) interactions (56) with Phe-10, known to be important for peptide-MHC binding (48). Thus, the dynamic movement by Tyr-84 in the G9GF-stretched structure might allow Phe-10 to be accommodated by the MHC F-pocket (Fig. 6*E*) rather than forming a more prominent central bulge, observed in most other MHCI structures with longer (>9 residues) peptides.

## Discussion

The NOD mouse model of T1D is an important tool for investigating the role of T-cells in the destruction of islet β-cells in the pancreas. Other molecular investigations of T-cell-induced autoimmunity have shed light on the selection and mode of action of autoreactive T-cells. For example, we have recently demonstrated that a preproinsulin-specific human TCR derived from a CD8^+^ T-cell bound with extremely weak affinity and a highly focused binding footprint (5). Other studies of autoreactive T-cells in other disease models have also demonstrated suboptimal TCR binding, either through weak TCR affinity (20), poor pMHC stability (18), topologically unusual TCR binding (19, 20), or a combination (5, 20). These observations have led to the suggestion that autoreactive T-cells receive weak or unconventional signals in the thymus that lead to positive selection rather than deletion. Here, we show that the G9C8 T-cell clone is reactive to an autoantigenic peptide that is part of the insulin protein but that the native epitopes were both relatively unstable compared with a heteroclitic peptide with optimal anchor residues. The TCR from this clone bound with weak affinity to the native epitopes, resulting in lower functional avidity. This combination adds support to the notion that selection of this clone could occur through weak T-cell signaling in the thymus. T-cells that have high affinity TCRs for more stable insulin-derived epitopes would probably be deleted through negative selection, explaining their absence in the periphery. The high levels of insulin expressed by β-cells and the probable high levels of G9G/G9GF epitopes on the surface of these cells might bridge the activation threshold of G9C8-like T-cells, inducing the autoreactivity observed. Surprisingly, despite a weak monomeric affinity for the G9C8 TCR, H-2K^d^·G9G tetramers could still robustly identify cognate T-cells. It is possible that the comparatively strong murine pMHC-CD8 affinity, compared with human pMHC-CD8 (57, 58), could play a role in stabilizing this weak affinity interaction at the cell surface (59, 60). Importantly, the G9C8 T-cell did not express an inherently weak binding TCR, because peptide substitution of Gly to Val at position 9 resulted in anti-viral-like affinity (10, 12, 13). The corresponding enhanced tetramer staining using the G9V peptide paves the way for the development of improved reagents to isolate, phenotype, and clonotype insulin-reactive CD8^+^ T-cells to better follow and determine their role in disease progression. Furthermore, this demonstration that the G9C8 TCR could bind to an altered ligand with >10 times higher affinity compared with the native ligands opens up the intriguing possibility that this T-cell clone could potentially be primed by a more immunogenic target and then cross-react with insulin B chain epitopes expressed by β-cells through a molecular mimicry type mechanism. Thus, this altered ligand could also be used to test the potential role of molecular mimicry on disease outcome.

The stability of the pMHC complex is critical in the presentation of epitopes to T-cells, because unstable pMHC will be present at lower concentrations or absent on the surface of antigen-presenting cells. To confound this issue, previously (49, 61, 62) and here, we found that modifications that altered pMHC stability also had a large effect on TCR binding affinity. H-2K^d^ is unusual compared with the binding motif for most other mouse alleles (that have an anchor at position 5 and the C terminus) in that it anchors at positions 2 and the C terminus, reminiscent of most human peptide-MHC binding motifs. Thus, our observations concerning the effects of TCR binding affinity upon altering the C-terminal anchor could be unique to this murine MHC allele. Because the peptide modifications were located at the C terminus, and the most solvent exposed peptide residues were Glu-7 and Arg-8, it is reasonable to speculate that the G9C8 TCR focuses on the C terminus of the peptide. This is consistent with our previous data demonstrating that modification of these residues (particularly Arg-8), along with Val-4, which was also pointing out of the groove according to our structural analysis, reduced T-cell activation (32, 52). This observation could explain the strong binding affinity between the G9C8 TCR and G9V, because Val-9 might stabilize the main TCR-peptide contact region, enabling more optimal contacts. Binding to this region of the peptide may also explain why, although the G9C8 TCR bound with a similarly weak affinity to H-2K^d^·G9G and H-2K^d^·G9GF, the G9C8 T-cell was more sensitive to the G9G peptide. The unusual presentation mode of the G9GF peptide may affect the dynamics of TCR binding, perhaps altering the formation of an optimal immune synapse or inhibiting the formation of TCR catch bonds that have recently been shown to play an important role in T-cell activation (11).

Our structural investigations also revealed a novel and unexpected mode of peptide presentation that has far reaching implications for T-cell antigen recognition in general. Although structures of different length versions of the same peptide have been published before, this is the first example in which the peptide alters the shape of the MHCI binding groove to accommodate an extra residue in the F-pocket. Additional residues at the N terminus and C terminus have been shown to have the following effects: 1) the central bulge of the peptide was altered because the extra residue could not be accommodated by the closed N-terminal end of the MHC binding groove (53–55); 2) the 9-mer version of the peptide assumed the same conformation as the 10-mer version of the peptide by using peptide residue 1, rather than residue 2, as the anchor (63); or 3) extra residues protruded from the groove at the peptide termini (64, 65). Here, the C-terminal end of the 10-mer G9GF peptide formed a dynamic interaction with the MHC binding groove. H-2K^d^·G9GF crystallized with three molecules in the asymmetric unit, demonstrating two distinct conformations. The dominant conformation observed in two of the copies (G9GF-stretched) forced the MHC binding groove to open to accommodate the bulky side chain of Phe-10, resulting in MHC residue Tyr-84 swinging 8.2 Å and altering the shape of the MHC F-pocket. Usually, the central residues of longer peptides are squeezed into more extended conformations because of the closed nature of the MHCI binding groove, as observed in the G9GF-bulged model of the structure. In the G9GF-stretched structure, the movement around the MHC F-pocket enabled the C terminus of the G9GF peptide to slide further down the groove so that the N terminus of the peptide could adopt a potentially similar conformation to the G9G and G9V 9-mer peptides. The ability of the G9GF 10-mer peptide to “mimic” the conformation of the 9-mer peptides is likely to be an important factor facilitating recognition of the G9GF peptide by the G9C8 TCR. The dynamic nature of the MHC binding groove was highly unexpected and adds to other studies in which a distinct movement in the MHC helices and/or peptide has been observed (66, 67). Combined, these data provide important evidence demonstrating the highly flexible nature of peptide presentation by MHC. Interestingly, a previous study implemented mutation of Arg-84 for Ala-84 for the stable generation of a single chain pMHC (68). Our findings would suggest that this mutation could have a substantial effect on the shape and dynamics of the MHC F pocket, leading to potential changes in peptide presentation. The flexibility we observed around the F-pocket also has implications for so-called TCR-pMHC “catch bonds.” A recent study demonstrated that, under force, some TCR and pMHC interactions can become stronger, resulting in enhanced T-cell activation (11). The formation of catch bonds suggests that the TCR, pMHC, or both undergo structural rearrangements when under force during binding at the cell surface, explaining the increase in binding strength. Our data, demonstrating the potential dynamic nature of the region around the MHC F-pocket, fits well with the notion of catch bond formation.

In summary, we show that insulin reactivity by a CD8^+^ T-cell clone, known to induce T1D, is characterized by weak TCR affinity to a highly unstable pMHC. The G9C8 TCR was able to bind more strongly to a peptide altered at the C terminus, demonstrating the potential of this T-cell clone to be triggered by a more immunogenic target. This observation also suggests that the interaction between the TCR and pMHC is likely to be focused toward the C terminus of the peptide, explaining the difference in sensitivity between the C-terminally altered peptide ligands investigated. Finally, we demonstrate a novel mode of flexible peptide presentation in which the MHC can effectively “open the back door” to accommodate extra C-terminal peptide residues.

## Author Contributions

C. M., J. A. P., E. D. L., P. J. R., K. B., A. T., and D. K. C. performed experiments. P. J. R. and D. K. C. performed the structural analysis. A. K. S., F. S. W., and D. K. C. conceived and funded the study and wrote the manuscript.

## Footnotes

7 The abbreviations used are:

T1D: type 1 diabetes
TCR: T-cell receptor
pMHCI: peptide-major histocompatibility complex class I
NOD: non-obese diabetic
Bistris propane: 1,3-bis[tris(hydroxymethyl)methylamino]propane
HB: hydrogen bond
vdW: van der Waals interaction
β2m: β2 microglobulin.
